# Virtual Reality Emergency Tracheostomy Simulation: A Feasibility Study on Intensive Care Healthcare Professional Learning Outcomes

**DOI:** 10.7759/cureus.83172

**Published:** 2025-04-29

**Authors:** Benjamin Rowlands, Shruti Suresh, Sung Yeon Kwak, Jessica Brown, Shreiya Narayanan, Chris Jacobs

**Affiliations:** 1 Anaesthesiology, Walsall Manor Hospital, Walsall, GBR; 2 Critical Care Medicine, Great Western Hospitals NHS Foundation Trust, Swindon, GBR; 3 Medical Education, Great Western Hospitals NHS Foundation Trust, Swindon, GBR; 4 The Academy, Great Western Hospitals NHS Foundation Trust, Swindon, GBR; 5 Internal Medicine, Great Western Hospitals NHS Foundation Trust, Swindon, GBR; 6 Department of Psychology, University of Bath, Bath, GBR; 7 Postgraduate Medical Education, Great Western Hospitals NHS Foundation Trust, Swindon, GBR

**Keywords:** adult intensive care, simulation in medical education, simulation medicine, tracheostomy, tracheostomy complications, virtual reality simulation

## Abstract

Introduction: The ability to manage tracheostomy emergencies is vital within the intensive care unit (ICU); however, due to clinical pressures, access to teaching and training is often limited. Simulation training using portable virtual reality (VR) headsets may provide increased access to this training without significant time away from clinical duties. This study aims to evaluate whether the management of a tracheostomy emergency can be effectively delivered through VR simulation within a clinical environment. The study used Trachosim® software produced by Goggleminds®.

Methods: This study recruited 28 clinical professionals working within the ICU and utilised a single-group post-test observation design. All participants were asked to complete a validated survey, the Immersive Technology Evaluation Measure (ITEM), using an online questionnaire to assess different aspects of the utility of the educational intervention. The study used descriptive statistics to assess overall data and used the Mann-Whitney U test to compare doctors to allied health professionals.

Results: The simulation showed high levels of immersion, strong intrinsic motivation, and a high quality of debrief. The cognitive load was deemed moderate, and the system usability was above the acceptable threshold. There were no statistically significant differences between doctors and allied health professionals.

Conclusion: This study showed that Trachosim® has good potential for increasing and improving training around tracheostomy emergencies within the ICU. The results suggest that VR is an appropriate teaching intervention to increase simulation training accessibility within a clinical environment. Further work should be done to compare VR intervention to alternative teaching modalities, as well as long-term outcomes such as knowledge and skill retention and improved patient outcomes.

## Introduction

A tracheostomy is a surgically created artificial opening of the anterior neck into the trachea. Indications for this procedure include long-term ventilation and weaning, facial and upper airway trauma, prevention of aspiration, and airway protection [1]. The National Confidential Enquiry into Patient Outcome and Death (NCEPOD) in 2015 estimated that approximately 12,000 tracheostomies are performed annually in the UK [2]. Immediate complications are relatively uncommon, occurring in only 5.3% of surgical tracheostomies. Post-tracheostomy complications such as obstruction or displacement accounted for 60% of tracheostomy emergencies, seen in both the intensive care and ward environments, occurring in 21% and 24.3% of cases, respectively [3]. Unfortunately, while these complications are uncommon, they are known to carry high rates of morbidity and mortality [4].

At present, in the UK, every tracheostomy emergency should be managed using the National Tracheostomy Safety Project’s "green" algorithm [5]. Healthcare professionals, especially those who encounter surgical airways, must ensure that they are proficient in managing tracheostomy emergencies. Hence, there is a clear indication that the training of healthcare professionals in managing emergency tracheostomy care should be enhanced [6]. However, since tracheostomy emergencies are relatively rare, it is difficult for healthcare professionals to retain these skills at a sufficient level through clinical exposure alone. Therefore, it is important to find alternative ways of increasing exposure to these clinical events, such as simulation-based learning.

The benefits of simulation training have been well documented in graduate medical education. In particular, this learning environment allows learners to develop the skills needed to manage emergency situations in a controlled manner without the risk of patient harm [7]. Recently, virtual reality (VR) has emerged as a novel method of delivering simulation. VR offers benefits for learners and educators, delivering cost-effective, repeatable, standardised clinical training on demand [8].

Previous VR simulations for tracheostomy emergencies have highlighted that VR content focusing on decision-making, equipment recognition, and algorithmic learning can deliver positive educational results [9]. While VR has shown comparable effectiveness to face-to-face teaching, its limitations include technology issues and the requirement for facilitation by an individual with appropriate technical expertise and clinical knowledge [10].

Despite the well-documented effectiveness of simulation-based training, there are clear accessibility issues for those working within a busy clinical environment. This study aims to evaluate whether the management of a tracheostomy emergency can be effectively delivered through VR simulation within a clinical environment. It aims to assess the use of a "Tea Trolley" like teaching method, in which the simulation is performed within the intensive care clinical environment, rather than taking clinicians away from their daily schedule. While this does not require staff to leave their clinical duties, it does rely on the availability of a quiet side room or clinical space. Additionally, although each session lasted no longer than 20 minutes, including orientation and simulation, the total time commitment from staff should be considered when assessing feasibility for routine implementation.

## Materials and methods

Recruitment

From March to April 2024, 28 clinical professionals were recruited through convenience sampling from the Intensive Care Unit at Walsall Manor Hospital (NHS). This included doctors, nurses, physiotherapists and healthcare students. There were no exclusion criteria.

Study design

**Figure 1:**
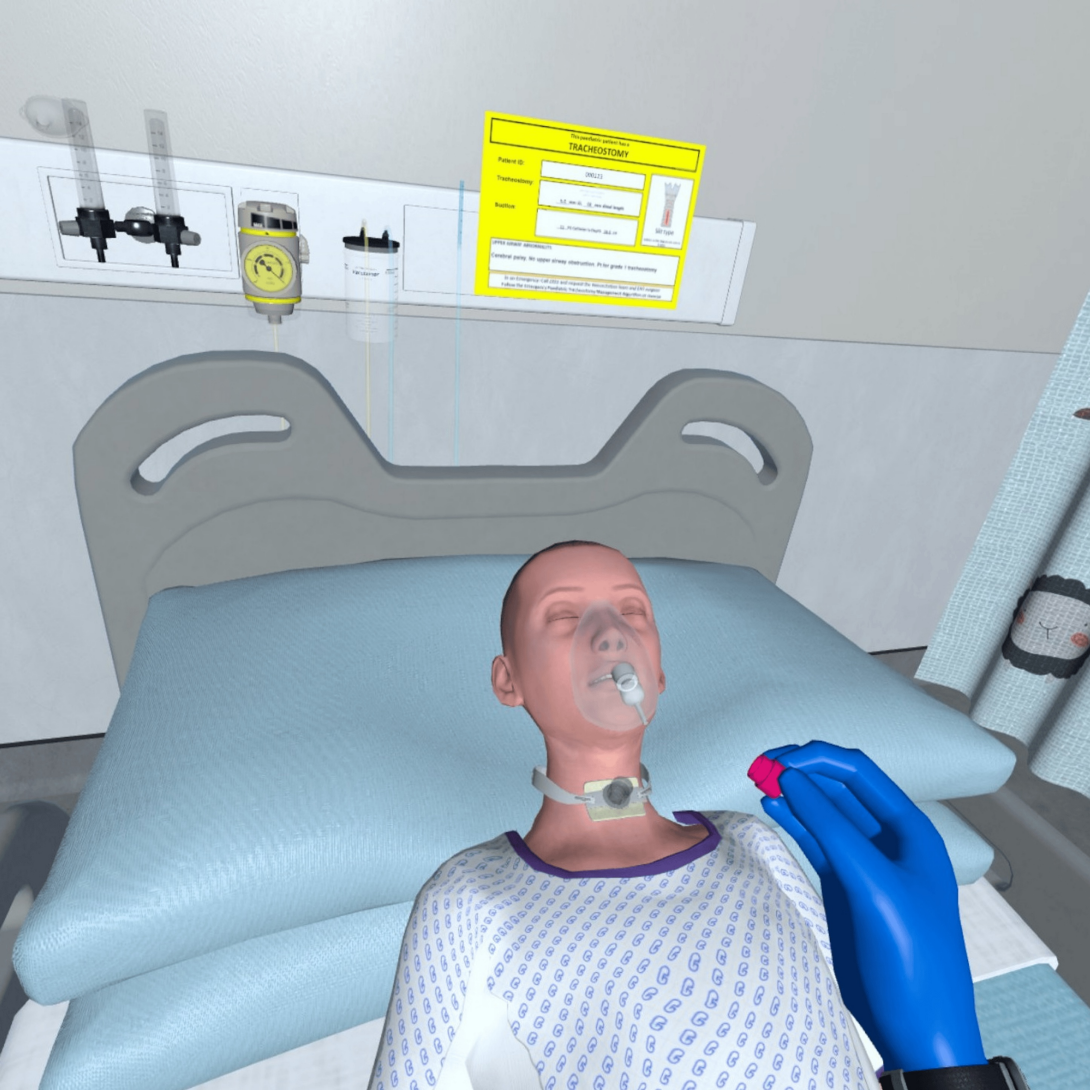
Example of Goggleminds® Trachosim® VR paediatric patient interactions Image created using Goggleminds® Trachosim® VR simulation; used with permission.

**Figure 2:**
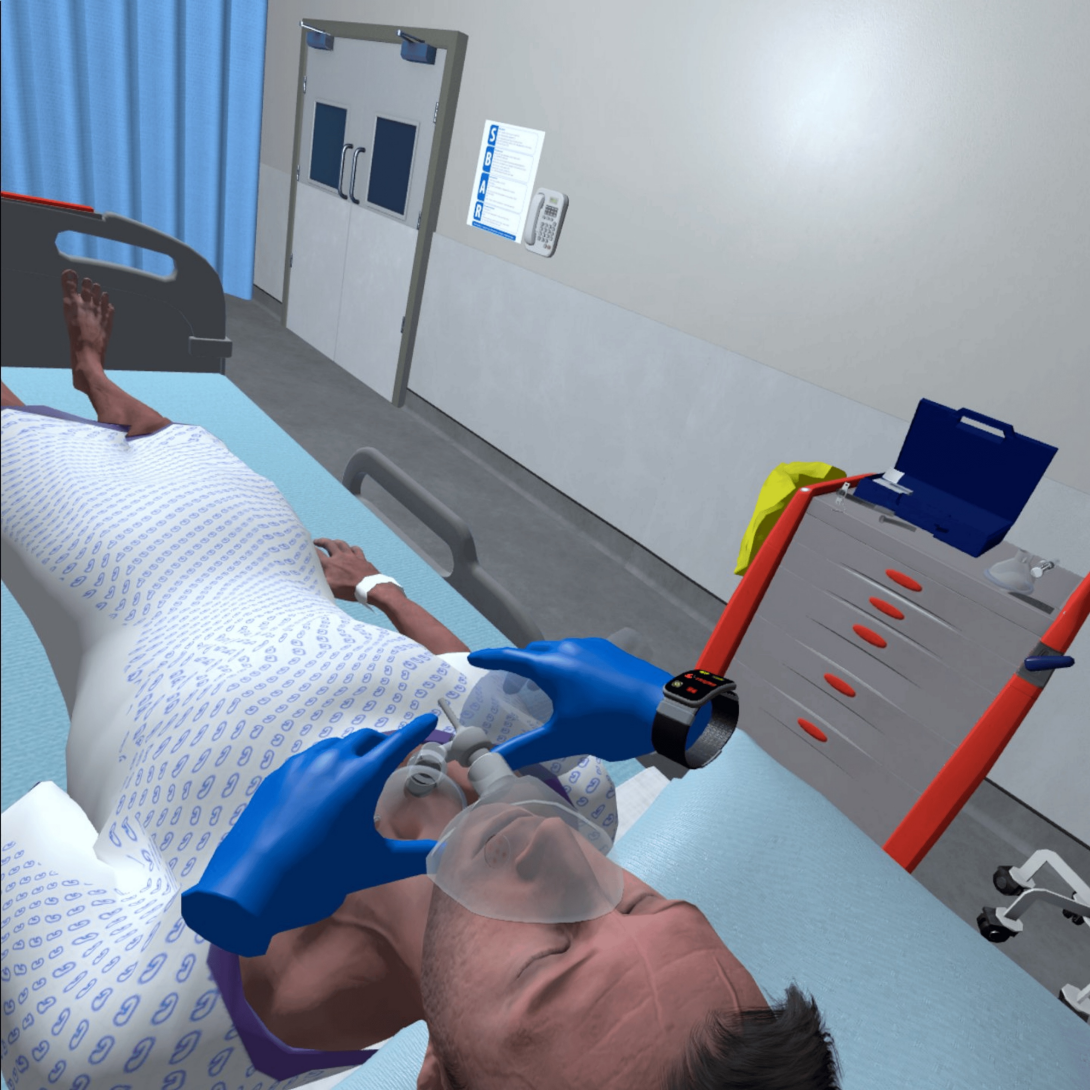
Example of Goggleminds® Trachosim® VR adult patient interactions Image created using Goggleminds® Trachosim® VR simulation; used with permission.

A single-group post-test observation design was employed. Participants were introduced to the Goggleminds® Trachosim® VR simulation and received a 15-minute orientation on how to operate the system. Following this, they progressed through the virtual simulation independently, with the internal software providing guidance as necessary and the researcher assisting with minor technical issues. The simulation required the participant to manage an adult patient with a blocked tracheostomy, following the "National Tracheostomy Safety Project - Emergency Tracheostomy Management" algorithm [5]. Example simulated patient interactions are shown in Figures 1, 2.

Data collection

After the simulation, participants were asked to complete the "Immersive Technology Evaluation Measure" (ITEM) using an online questionnaire. This assesses the overall acceptability of the teaching modality by focusing on five main domains: immersion, intrinsic motivation, cognitive load, system usability, and debriefing feedback [11].

Statistical evaluation

Descriptive statistics, including measures of central tendency and dispersion, were calculated. Data normality was assessed using the Shapiro-Wilk test, with a p-value < 0.05 indicating a non-normal distribution. Median values for both groups were evaluated and compared. The Mann-Whitney U test was used for group comparisons, with statistical significance set at p < 0.05. All statistical analyses and graphs were conducted using StatsDirect software (version 3.9.9, StatsDirect Ltd, Wirral, UK).

Ethics

Participation in the study was entirely voluntary, and informed consent was obtained for the inclusion of questionnaire data within the study. All data were anonymised to ensure confidentiality. According to the Medical Research Council and NHS Health Research Authority online tool, a formal NHS Research Ethics Committee review was not required. The participating NHS trust was selected as part of a quality improvement initiative to reduce errors in tracheostomy care.

## Results

Twenty-eight participants completed the Trachosim® VR educational simulation and completed the post-intervention questionnaire. Eleven were doctors (39%) and 17 were allied healthcare professionals (61%). The Shapiro-Wilk test indicated that the data for both groups did not follow a normal distribution (p < 0.05). There were no missing data. The outcomes for each of the Immersive Technology Evaluation Measure (ITEM) domains are summarised in Table 1.

**Figure 3:**
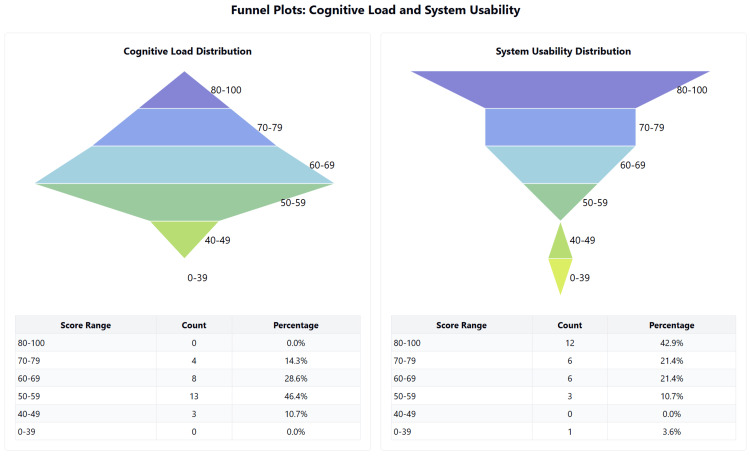
Distribution of scores for the Cognitive Load and System Usability domains of ITEM ITEM: Immersive Technology Evaluation Measure.

**Figure 4:**
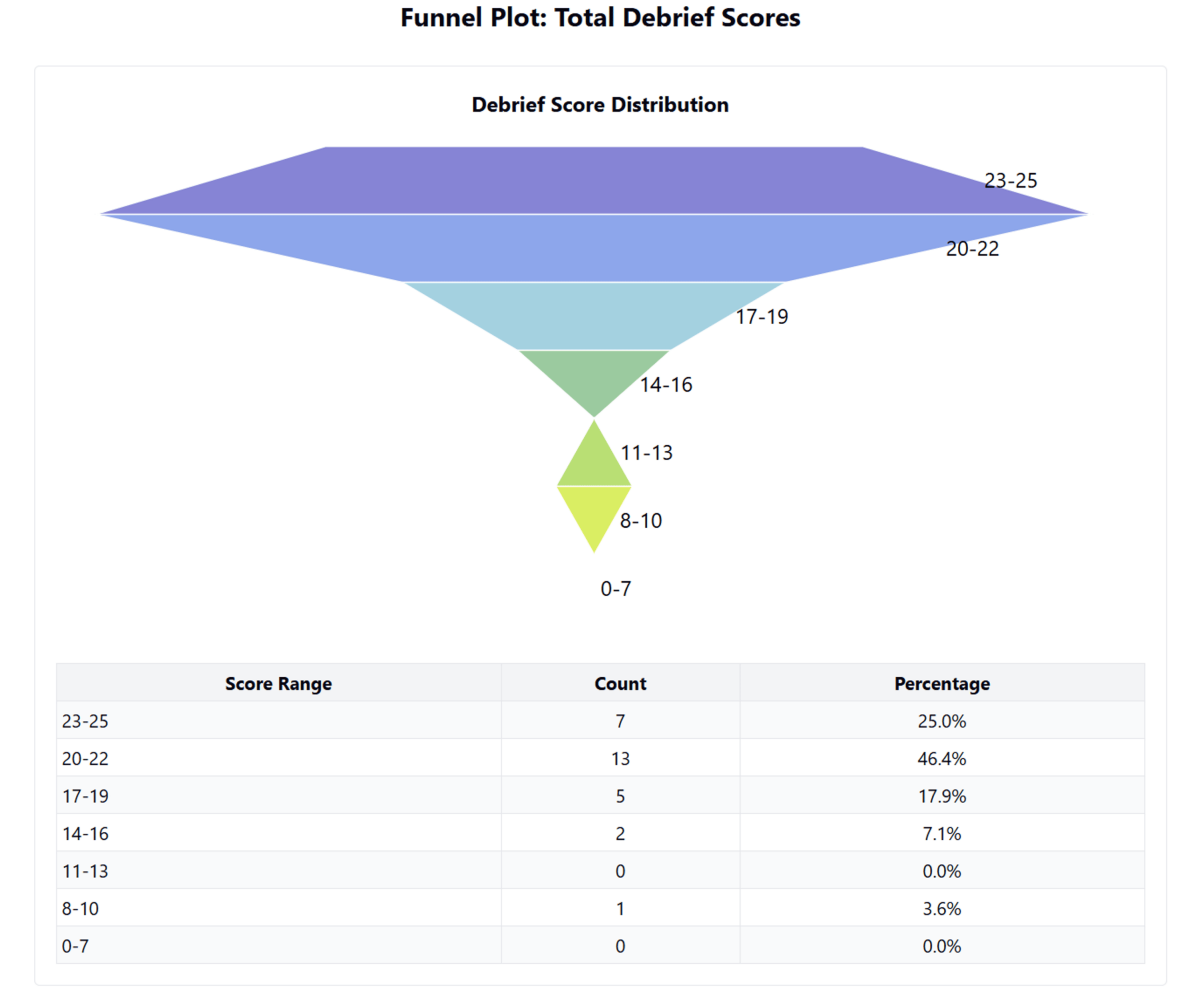
Distribution of scores for debrief domain of ITEM ITEM: Immersive Technology Evaluation Measure.

**Figure 5:**
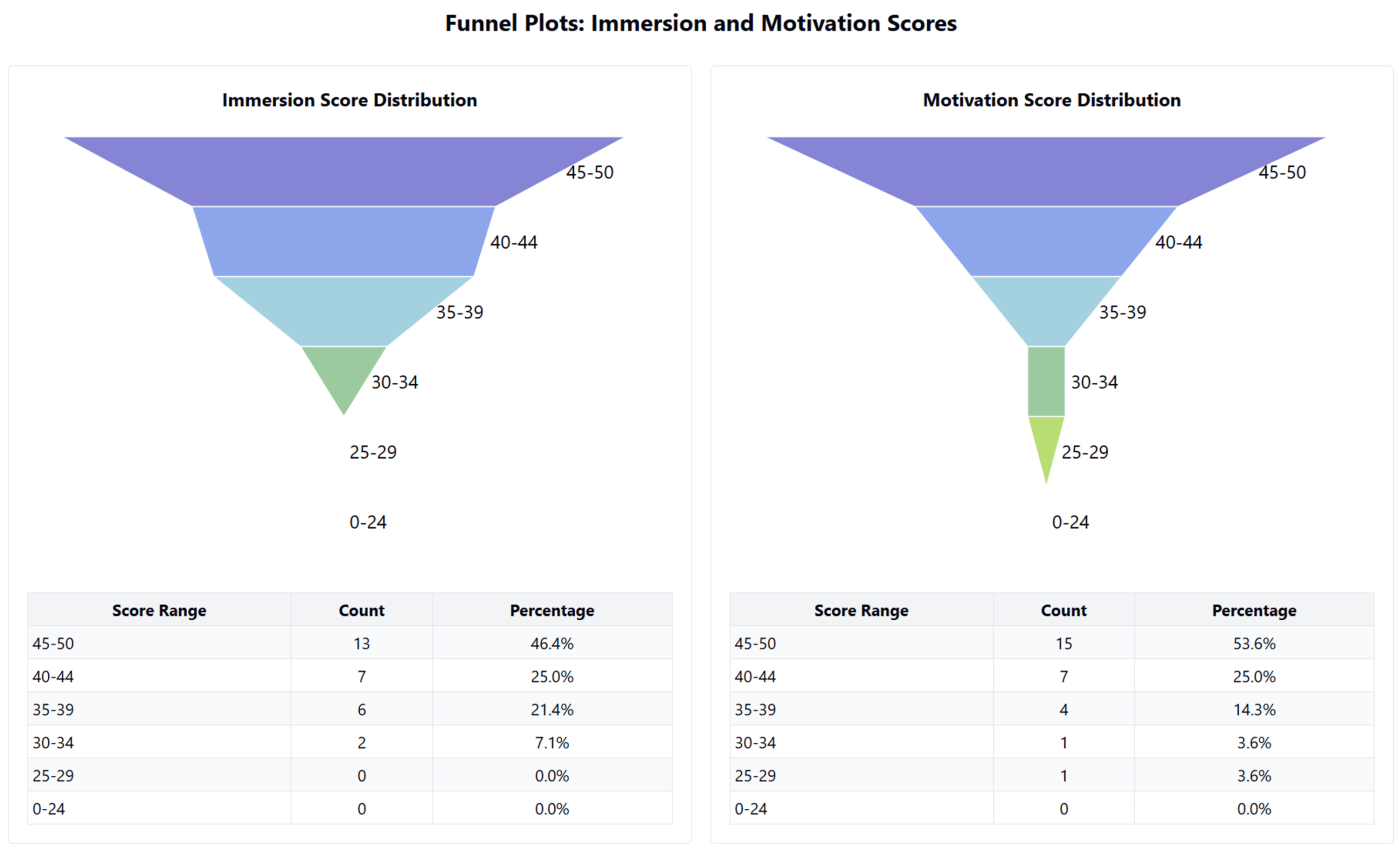
Distribution of scores for the Immersion and Motivation domains of ITEM ITEM: Immersive Technology Evaluation Measure.

The simulation reported a high level of immersion, with a median score of 43.5 (IQR: 39-45) out of 50, and strong intrinsic motivation, with a median score of 45 (IQR: 41-48) out of 50. System usability was rated with a median score of 73 (IQR: 66-89) out of 100, which is above the acceptable threshold of 68. Cognitive load, measured using the NASA TLX single score, had a median score of 59.9 (IQR: 53.3-68.3), indicating a moderate cognitive load on a scale of 100. The quality of the debrief, assessed using the adaptive PEARLS questionnaire, achieved a high median score of 20 (IQR: 19-22) out of 25. Figures 3-5 summarise these values as a box-and-whisker plot.

**Table 1:** Descriptive data summary of the ITEM 5 domains ITEM: Immersive Technology Evaluation Measure.

Variable	Total Immersion (max 50)	Total Motivation (max 50)	Total Cognitive Load (max 100)	Total System Usability (max 100)	Total Debrief (max 25)
Upper quartile	45	48	68.32	89	22
Median	43.5	45	59.97	73	20
Lower quartile	39	41	53.28	66	19
Interquartile range	6	7	15.04	23	3
Minimum	31	29	46.62	36	8

Group comparison

**Figure 6:**
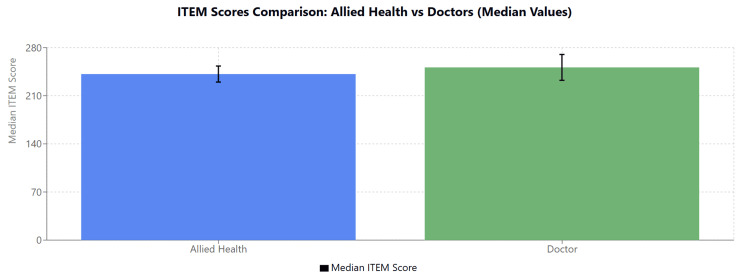
Total ITEM median and interquartile range (IQR) for doctors and AHPs ITEM: Immersive Technology Evaluation Measure, AHP: allied healthcare professional.

The median values for registered doctors and allied health professional staff were compared, with registered doctors showing comparable median values to those of allied healthcare professionals (AHPs) (see Table 2 and Figure 6).

**Table 2:** Descriptive data summary of the ITEM 5 domains for groups (doctors and AHPs) ITEM: Immersive Technology Evaluation Measure, AHP: allied healthcare professional.

Variable	Total Immersion Doctors (max 50)	Total Motivation Doctors (max 50)	Total Cognitive Load Doctors (max 100)	Total System Usability Doctors (max 100)	Total Debrief Doctors (max 25)	Total Immersion AHP (max 50)	Total Motivation AHP (max 50)	Total Cognitive Load AHP (max 100)	Total System Usability AHP (max 100)	Total Debrief AHP (max 25)
Maximum	45	49	76.59	96	25	50	50	79.92	96	25
Upper quartile	45	46	66.66	84	21	46	49	69.99	90	24
Median	44	44	56.61	74	20	42	45	63.33	72	21
Lower quartile	41	41	53.28	68	19	39	41	53.28	64	20
Interquartile range	4	5	13.38	16	2	7	8	16.71	26	4
Minimum	37	36	49.95	58	16	31	29	46.62	36	8

Results by measure

No significant differences were found between doctors and AHPs across any of the measured parameters. All p-values were greater than 0.05, indicating that observed differences between the groups were not statistically significant.

Immersion

The Mann-Whitney U test comparing immersion levels between doctors (median = 44) and AHP staff (median = 42) yielded U = 91.5, U' = 95.5, with a two-sided p-value of 0.935. The median difference was 0 (95.31% CI: -3 to 5). These results indicate that immersion scores were nearly identical between the two professional groups.

Intrinsic motivation

The Mann-Whitney U test comparing intrinsic motivation between doctors (median = 44) and AHP staff (median = 45) yielded U = 75.5, U' = 111.5, with a two-sided p-value of 0.409. The median difference was -2 (95.31% CI: -5 to 3). While AHP staff showed a slightly higher median motivation score, this small difference was not statistically significant.

Cognitive load

The Mann-Whitney U test comparing cognitive load between doctors (median = 56.61) and AHP staff (median = 63.33) yielded U = 77, U' = 110, with a two-sided p-value of 0.448. The median difference was -3.33 (95.31% CI: -10.05 to 3.33). Although AHP staff reported numerically higher cognitive load than doctors, this difference did not reach statistical significance.

System usability

The Mann-Whitney U test comparing system usability between doctors (median = 74) and AHP staff (median = 72) yielded U = 94.5, U' = 92.5, with a two-sided p-value of 0.972. The median difference was 0 (95.31% CI: -12 to 12). Both professional groups rated the system usability very similarly, with no detectable difference between groups.

Debrief

The Mann-Whitney U test comparing debrief scores between doctors (median = 20) and AHP staff (median = 21) yielded U = 73, U' = 114, with a two-sided p-value of 0.339. The median difference was -1 (95.31% CI: -3 to 1). Debrief scores were slightly higher in the AHP group, but this difference was not statistically significant.

## Discussion

VR simulation has recently emerged as a safe and interactive educational platform, especially for training in rare, high-risk scenarios such as tracheostomy emergencies that are otherwise challenging to practise in the real-world clinical setting. A study by Lateef et al. demonstrated that VR simulation reduces errors and builds procedural confidence by allowing repetitive, deliberate practice without patient risk [12]. Furthermore, VR offers portability and scalability, aligning with on-demand training approaches like the "tea trolley" method [13]. It also provides a high level of fidelity that helps bridge the gap between theoretical learning and clinical practice, making it an invaluable tool for healthcare education. Cheng et al. highlighted that technical issues, including malfunctions and the reliance on facilitators for troubleshooting, can disrupt the learning process in VR-based training [14]. Despite its potential long-term cost-effectiveness, the high costs of initial setup and maintenance of VR systems can be prohibitive [15].

This study was conducted to evaluate the efficacy of Tracheosim in managing tracheostomy-related emergencies by doctors and AHPs in the ICU. The effectiveness of the modality was assessed using the ITEM questionnaire, which focused on five particular domains: cognitive load, intrinsic motivation, immersion, system usability, and debriefing.

ITEM 5 domains

Immersion is vital to VR simulation as it enables a suspension of belief and simultaneously creates psychological safety. Studies have found that creating consistent learning simulations allows for the evaluation of learner performance and knowledge [16]. On the other hand, intrinsic motivation has been found to be the strongest predictor for educational outcomes of learners, in the context of attention to subject matter, effort generated, and changes to behaviour and grades [17]. The study demonstrated high levels of immersion and intrinsic motivation, suggesting that participants found the platform engaging and were internally driven to learn. These factors are crucial for experiential learning, as described by Kolb's experiential learning theory, which emphasises active participation and reflection to solidify knowledge. The combination of these two domains underscores the importance of fidelity, as a high-fidelity environment promotes task relevance and learner engagement. This is particularly important for the participants of our study who operate in an intensive care setting and might face low-frequency but high-risk scenarios such as tracheostomy emergencies. The high-fidelity environment of VR simulation provides a safe and engaging platform for repeated practice, making it an ideal modality to help these professionals maintain preparedness for critical events.

Cognitive load is based on the assumption that learners only have a limited amount of working memory available to them during the learning process, but also have infinite long-term memory formed by cognitive maps (schemas) [18]. The Cognitive Load Theory proposes that learning is most effective when instructional design reduces extraneous load (unnecessary mental effort) and instead focuses on intrinsic load (task complexity) and germane load (processing to deepen understanding) [19]. In the context of this VR simulation, the extrinsic load was mitigated through the availability of resources such as the emergency tracheostomy algorithm and VR equipment demonstration. In contrast, the intrinsic load was greater as learners required pre-existing knowledge of tracheostomy anatomy, pathophysiology, and the high task burden. The moderate cognitive load demonstrated could therefore be explained by a higher intrinsic load compared to extraneous load, which could be attributed to the difference in skill set of the participants.

Debriefing in healthcare is a process by which reflective learning is achieved through guided conversation [20]. The high debrief score suggests that participants found the debriefing process very effective. The study used the PEARLS (Promoting Excellence and Reflective Learning in Simulation) framework, which emphasises a structured yet flexible approach that integrates learner self-assessment, focused facilitation, and directive feedback. Schön’s concept of reflection-on-action through retrospective contemplation encourages critical analysis of an experience after it has occurred to draw insights for future practice [21]. After completing the tracheostomy emergency simulation, participants were encouraged to reflect on their actions, decisions, and adherence to the algorithm. This allowed them to evaluate what went well and identify areas for improvement, fostering deeper understanding and skill refinement. Evidence of this learning could further be inferred by a demonstrable difference in clinical judgement, reasoning, and decision-making with repeated practice using Trachosim®.

The system's usability influences the participant's experience and cognitive mechanisms, thereby directly and indirectly affecting learning [22]. The SUS measured exceeded the benchmark for acceptable usability. This suggests that participants generally found the VR simulation intuitive and user-friendly, which could thereby decrease extraneous cognitive load. However, the interquartile range of 66 to 89 indicates some variability in user experiences, with a few participants potentially facing difficulties. High system usability is critical for boosting user satisfaction and allowing learning to be focused on educational content rather than struggling with the interface. Furthermore, usability is essential for acceptance and scalability, and so a less intuitive system that requires extensive facilitation may limit independent, widespread use [16].

Differences between doctors and AHPs

The results showed minor differences in the ITEM domains between doctors and AHPs, although none were statistically significant. Doctors reported slightly higher immersion and system usability scores, likely due to greater familiarity with similar clinical scenarios and exposure to similar training tools or technology in their professional development. AHPs exhibited higher intrinsic motivation, likely reflecting their perception of the training as more novel and directly beneficial. Also, their higher debrief scores suggest that they gained significant value from the structured feedback, which helped clarify uncertainties and reinforce key learning points. These findings underscore the importance of tailoring sessions to address the specific needs of diverse professional groups.

Limitations

This study also has some limitations that should be considered. The small sample size of 28 participants from a single NHS trust restricts the diversity of experiences and backgrounds, therefore limiting the generalisability to wider healthcare settings. It employed a single-group post-test observational design, which limits the ability to draw inferences between VR simulation and other training methods. Without a pre-test assessment, the study could not evaluate improvements in participant knowledge, skills, or confidence due to the VR training. Due to the independent nature of the simulation, the educational intervention is unable to assess the role that non-technical skills, such as teamwork and communication, would play in such an emergency. Lastly, the study also depended on researcher assistance for minor technical issues, highlighting potential challenges in implementing VR independently without dedicated facilitators.

Recommendations/future steps

The impact of the study can be further expanded by integrating VR simulation with the "tea trolley" teaching method. This approach would allow portability and on-demand accessibility, thereby enhancing practical learning opportunities in clinical settings. Additionally, conducting a multi-centre study across multiple NHS sites with a larger and more diverse cohort over an extended period would improve the applicability and statistical power of the findings. Future research should explore longitudinal outcomes, such as skill retention over time and real-world application in clinical settings. This could involve follow-up assessments at regular intervals to measure skill decay or persistence and determine whether VR-trained professionals maintain their proficiency when faced with actual emergencies. Also, studies should explore whether VR training translates into improved patient outcomes, faster response times, and better adherence to emergency protocols in practice.

## Conclusions

Trachosim® as an extended reality modality offers the opportunity to develop clinical skills in high-risk, low-frequency events such as tracheostomy emergencies. The study demonstrated high immersion and intrinsic motivation promoted by a high-fidelity environment that requires active participation and therefore encourages learning in a psychologically safe manner. While there is a moderate cognitive load attributed to aspects of intrinsic load, these could be ascribed to a skill set difference amongst participants. To reduce cognitive load, learning from VR simulation could be reinforced by face-to-face teaching sessions, which incorporate factual knowledge to develop skills required for higher-order thinking. Although there are limitations to this study, such as a small sample size and the requirement for a dedicated facilitator, Trachosim® usability and portability reflect its promising use as an acceptable and feasible model for learning.
